# Exploring the Relationship between Neuroticism and Perinatal Depressive Symptoms: Findings from a 2-Year, Multicenter Study in Italy

**DOI:** 10.3390/brainsci14040366

**Published:** 2024-04-09

**Authors:** Melania Severo, Annamaria Petito, Antonio Ventriglio, Salvatore Iuso, Giulio Ianzano, Alessia Marconcini, Elisa Giannaccari, Giuseppe Luigi Palma, Mario Altamura, Felice Sorrentino, Giuseppe Maruotti, Luigi Nappi, Antonella Caroli, Antonello Bellomo

**Affiliations:** 1 Department of Humanistic Studies, University of Foggia, 71122 Foggia, Italy; melania.severo@unifg.it (M.S.); salvatore.iuso@unifg.it (S.I.); 2 Department of Clinical and Experimental Medicine, University of Foggia, 71122 Foggia, Italy; annamaria.petito@unifg.it (A.P.); giulioianzano@tiscali.it (G.I.); mario.altamura@unifg.it (M.A.); antonello.bellomo@unifg.it (A.B.); 3 Unit of Gynaecology, “San Paolo” Hospital, 70123 Bari, Italy; marlessia@hotmail.com; 4 Unit of Gynaecology, “Vito Fazzi” Hospital, 73100 Lecce, Italy; e.giannaccari@gmail.com; 5 Unit of Psychology, “Vito Fazzi” Hospital, 73100 Lecce, Italy; giuseppeluigi.palma@gmail.com; 6 Department of Medical and Surgical Sciences, University of Foggia, 71122 Foggia, Italy; felice.sorrentino.1983@gmail.com (F.S.); maruottig@gmail.com (G.M.); luigi.nappi@unifg.it (L.N.); 7 Department of Health Promotion, Regione Puglia, 70121 Bari, Italy; a.caroli@regione.puglia.it

**Keywords:** neuroticism, personality, perinatal depression, pregnancy, EPDS

## Abstract

Neuroticism is a personality trait associated with the risk of affective disorders and perinatal depression. We investigated the relationship between different levels of neuroticism, psychological characteristics, and depressive symptoms in a sample of pregnant women (N = 2631) who accessed the gynecology departments in the Puglia Region (Italy) from July 2020 to November 2022. Women were assessed for depressive symptoms and associated risk factors in their third trimester of pregnancy (T0) and after childbirth (T1), and followed-up at 6 months and 1 year after delivery if presenting signs of depression (T2–T3). The Edinburgh Postnatal Depression Scale (EPDS) was used to screen depressive symptoms, and neuroticism was assessed through the subscales of the NEO Five Factor Inventory. Standardized measures of resilience, coping strategies, partner attachment, and quality of life were also employed. Higher levels of neuroticism were significantly associated with: (a) higher scores on the EPDS; (b) higher anxiety in the experience of close relationships; (c) lower psychological wellbeing; (d) lower levels of resilience; (e) lower levels of active coping; and (f) higher levels of self-blame. Our findings may suggest that neuroticism is a specific associated factor of perinatal depression and should be routinely assessed in the clinical screening of pregnant women in order to promote an early referral to psychological or psychiatric support services.

## 1. Introduction

An early detection of depressive symptoms during pregnancy and postpartum is crucial for the healthy outcome of pregnancy as well as maternal, newborn, and child health [1,2,3,4]. San Martin Porter et al. reported that women with positive scores at the screening program for depressive symptoms in the peripartum (Queensland Study) also have increased odds of a spontaneous onset of labor and decreased odds of an operative delivery [1]. Reilly et al. reviewed the available evidence from the literature and found that women enrolled in screening programs for the detection of perinatal depression were successfully referred to specific services with relevant benefits in terms of parenting outcomes [2]. Accordingly, the international clinical guidance on the prevention of perinatal depression recommends a routinely appropriate screening of mood symptoms among pregnant women in order to safeguard their general pregnancy outcomes [3,4].

A large number of studies have tested the specific associations between psychological characteristics, personality traits, and the risk for women of developing perinatal depressive symptoms [5,6,7,8,9,10,11]. Here, we will focus on neuroticism as a specific personality trait supposed to be involved in the vulnerability model of mood disorders. Originally introduced and defined by Eysenck [12] in 1967, neuroticism is considered an endophenotype of genetic predisposition to develop affective disorders [13]. In fact, some genetic factors putatively influencing the individual variations in neuroticism substantially overlap with the candidate genes identified with internalizing disorders (e.g., gene encoding for the serotonin transporter, the brain-derived neurotrophic factor [BDNF]) [14]. Specifically, neuroticism is also identified as a personality factor predisposing individuals to negative emotions including fear, confusion, guilt, regret, or susceptibility to psychological stress [15]. Also, high levels of neuroticism underpin emotional instability, negative outlook, difficulty in adapting, poor self-control, a tendency to complain about life, and the inability to cope with severe psychological stress [13]. In fact, individuals reporting higher levels of neuroticism may experience ineffectiveness in coping with stressful situations and often may engage in worrying, rumination, or emotional avoidance [16]. An increased interest in neuroticism has been registered in recent decades [17], and several authors have considered this variable as an important predictor of depression in subjects exposed to stressful situations including pregnancy and the postpartum period [5,9,18]. The perinatal period may be a source of significant stress for many women, above all after childbirth or during the first few months of the newborn’s life. Thus, it has been argued that relevant levels of neuroticism may predispose women to an inadequate adjustment in these critical months, possibly leading to the onset of depressive symptoms during pregnancy as well as after childbirth [5,19]. Here we report that postpartum depression is a mood disorder occurring within 4 weeks to 1 year after delivery, including physical, emotional, and behavioral symptoms [9]. In addition, perinatal depression is defined as a clinical condition based on a cluster of depressive symptoms occurring in the course of pregnancy or after childbirth, triggered by low levels of education, low income, poor partner and social support, or co-occurring stressful life events [9]. Puyané and colleagues [19] have argued that certain personality traits, in particular neuroticism, are often associated with an increased vulnerability to postpartum depression. Iliadis et al. specified that pregnant women with higher levels of neuroticism were four times more likely to develop symptoms of depression than those with low levels of neuroticism [20]. However, it may be questionable whether neuroticism represents a predictive factor or a marker of depression among pregnant women. Some authors have suggested considering individual levels of neuroticism in order to estimate the risk of developing emotional or mood disorders (higher levels are associated with higher risk), whereas some others have suggested that neuroticism may be a specific, identifiable pattern in patients suffering from mood disorders [5,6]. Bright et al. [21] also reported that higher levels of neuroticism could predict suicidal ideation among women during the perinatal period. In addition, neuroticism was significantly associated with low perceived social support during pregnancy as well as poor adjustment after childbirth [22,23]. Specifically, Chen and colleagues [24] suggested that social support and women’s sleep quality both acted as mediating factors of the indirect relationship between neuroticism and perinatal depression. Other significant associations have been found between higher levels of neuroticism and the individual insecure attachment style, lower levels of resilience, lower quality of life, and negative emotion-focused coping styles [25,26,27,28]. It might be concluded that neuroticism, as an enduring individual vulnerability, influences adjustment processes in general as well as interpersonal perceptions, emotional states, and the coping strategies used to manage stressful events. Thus, neuroticism should be considered a personality trait with poor variability in the course of life, and its assessment does not require a follow-up over time. Interventions and preventive strategies for the prevention of perinatal depression should be particularly focused on this variable.

The purpose of our study was to test the associations between a set of psychological characteristics (partner attachment, coping strategies, quality of life, and levels of personal resilience) as well as depressive symptoms (measured with the EPDS) of pregnant women with different levels of neuroticism (classified as low, medium, and high level). We hypothesized that women showing higher levels of neuroticism might present a higher number of depressive symptoms during pregnancy and postpartum. Also, women with higher baseline neuroticism might show more anxious and avoidant attachments to their partners, lower quality of life, more dysfunctional coping strategies, and lower levels of personal resilience. In line with the suggestions from the literature discussed, our study aimed to test these associations between neuroticism and other psychological characteristics in a large real-world setting.

## 2. Materials and Methods

### 2.1. Sampling and Study Design

We analyzed a convenience sample from a large screening and prevention program for perinatal depression conducted among pregnant women who accessed the gynecology departments in the Puglia Region, southern Italy, from July 2020 to November 2022. The sample included 2631 pregnant women in their third trimester of pregnancy, enrolled during pre-admission at the gynecology units of Policlinico Riuniti of Foggia/University of Foggia (Foggia, Italy), Vito Fazi Hospital (Lecce, Italy), and Di Venere Hospital (Bari, Italy). At intake (T0), the women underwent psychological screening. The initial screening included the following measures (as properly described in the Section 2.3): sociodemographic variables, the Edinburgh Postnatal Depression Scale (EPDS), the N scale of the NEO Five-Factor Inventory, the Experience in Close Relationships scale (ECR), the Connor–Davidson Resilience Scale (CD-RISC), the Brief-COPE, and the World Health Organization Quality of Life-Brief Version (WHOQOL-BREF). All pregnant women accessing the gynecology departments were screened. Underage women (<18 years old) and those unable to understand and communicate in Italian were excluded because of the lack of valid informed consent. Participation was free of charge, and the study was conducted in accordance with the World Medical Association’s Declaration of Helsinki and its subsequent revisions [29].

Subsequently, women were all re-evaluated with the EPDS within 7 days after childbirth, and those considered at risk of depression (EPDS total score ≥ 12) were re-evaluated with the EPDS at 1 and 6 months after childbirth. Notably, our screening was also configured as a primary and secondary prevention program, since women found to be at risk of perinatal depression were promptly informed and referred to specific territorial services, including family counseling and psychological services.

### 2.2. Project Authorization and Ethical Approval

This study is part of a regional project proposed by the Department of Health Promotion of the Apulia Region entitled “Governance of care for people in frailty”, ethically approved by special deliberation No. 65, issued on 12 March 2019, and funded by the Apulia Region through two deliberations (DGR No. 1392, issued on 2 August 2018, and DGR No. 2294, issued on 11 December 2018). The program was implemented by the University of Foggia (Unit of Psychiatry, Foggia, Italy) in collaboration with the gynecology units of Policlinico Riuniti di Foggia/University of Foggia (Foggia, Italy), Vito Fazi Hospital (Lecce, Italy), and Di Venere Hospital (Bari, Italy).

### 2.3. Measures

#### 2.3.1. Neuroticism Personality Trait

Neuroticism was evaluated using the N scale of the 60-items NEO Five-Factor Inventory (60-items NEO-FFI) by McCrae and Costa [15]. This instrument includes 60 items and explores five basic personality factors, namely neuroticism (N), agreeableness (A), conscientiousness (C), extraversion (E), and openness (O) (e.g., item 1 “*I am not a caring person*”; item 6 “*I often feel inferior to others*”) [15]. We employed the Italian translation of Subscale N, based on 12 items scored on a 5-point Likert scale ranging from 0 to 4, which describes neuroticism, defined as a basic personality trait associated with higher levels of anxiety, depression, and emotional lability (worry, fear, anger, frustration, loneliness, etc.). Higher scores represent higher levels of neuroticism. The mean score registered in healthy adult women of the general population was 16.77 ± 7.91, according to McCrae & Costa [15]. The 60-items NEO-FFI shows an equivalent factorial structure, reliability, and validity to the NEO Five-Factor Inventory [15,30]. It has also been translated into several languages and has demonstrated validity, reliability, and utility in a variety of contexts and cultures [15,31]. In addition, its internal consistency was confirmed in several studies [15,30,31].

#### 2.3.2. Depressive Symptoms

Depressive symptoms were assessed employing the Edinburgh Postnatal Depression Scale (EPDS). The EPDS is the most widely used instrument to assess the presence and severity of depressive symptoms in the postpartum period, with satisfactory psychometric characteristics of sensitivity and specificity in different cultures. The EPDS was proposed by Cox and Holden [32] in 1987 and assesses depressive symptoms during the last 7 days of observation. The test is self-completed and consists of 10 items, with a response scale ranging from 0 to 3 (total score range 0–30), with increasing values indicating greater severity (e.g., item 8 “*I felt sad and unhappy*”; item 9 “*I was so unhappy than I even cried*”). A total score ≥ 12 is considered as indicative of risk of depression, with sensitivity of 56 percent, specificity of 99 percent, and a positive predictive value of 91 percent [32]. The EPDS is considered a validated, standardized, and reliable tool for the screening of depressive symptoms but is not employed for diagnosing depressive disorders [32]. In this study, the Italian version validated by Benvenuti [33] was adopted, which shows an excellent internal validity (Cronbach’s alpha 0.79).

#### 2.3.3. The Experience in Close Relationships Scale

The Experience in Close Relationships scale (ECR) proposed by Brennan et al. [34] is a self-report instrument consisting of 36 items assessing individual differences with respect to attachment-related anxiety and avoidance in an attachment relationship. Respondents assign a rating to each item on a 7-point Likert scale from 1 (completely false) to 7 (completely true) (e.g., item 1 “*I prefer not to show my partner how I feel inside*”; item 2 “*I am afraid of being left*”). Higher scores indicate higher levels of anxiety or avoidance. The questionnaire shows a high level of validity and internal consistency. In this study, we employed the Italian version validated by Picardi et al. [35].

#### 2.3.4. Levels of Resilience

The Connor–Davidson Resilience Scale (CD-RISC) [36] is one of the most widely used scales to measure resilience. The authors of this scale defined resilience as a measure of coping skills in response to adversity and stress. This scale includes 25 items, each based on a 5-point Likert scale ranging from 0 to 4. Thus, the total score can range from 0 to 100 (e.g., item 1 “*I am able to adapt to change*”; item 2 “*I have stable and secure relationships*”). The higher the score, the greater the level of resilience achieved by the patient/individual. The Italian CD-RISC reports a good internal consistency and measurement stability.

#### 2.3.5. Coping Strategies

The Brief-COPE is a 28-item self-report questionnaire designed to measure effective and ineffective coping styles in dealing with a stressful life event. The scale identifies individuals’ main coping styles, scoring on the following domains (subscales): self-distraction, denial, substance use, behavioral disengagement, emotional support, venting, humor, acceptance, self-blame, religion, active coping, use of instrumental support, positive reframing, and planning. Each subscale consists of 2 items scoring on a Likert scale from 1 (*I habitually do not do this at all*) to 4 (*I habitually do just this*); consequently, the total scores on each scale range from 2 to 8 (e.g., item 1 “*I apply myself to work or other substitute activities to divert my mind from events*”; item 2 “*I focus my efforts on doing something about the situation I am in*”). Higher scores indicate higher use of a specific coping strategy. The psychometric characteristics of the Brief-COPE were found to be satisfactory, and the instrument reported a documented validity with an internal reliability and factor structure remarkably similar to that of the extended COPE [37]. In our study, we used the Italian version provided by Conti [38].

#### 2.3.6. Quality of Life

The Italian version of the WHOQOL-BREF (World Health Organization Quality of Life-Brief Version) is a 26-item instrument, proposed and validated by de Girolamo et al. [39], consisting of four domains: physical health (7 items), psychological health (6 items), social relationships (3 items), and environmental health (8 items); it also contains items on the quality of life and general health (e.g., item 5 “*How much do you enjoy life?*”; item 7 “*Can you concentrate on the things you do?*”). Each individual item of the WHOQOL-BREF is scored from 1 to 5 on a response scale, which is stipulated as a five-point ordinal scale. The WHOQOL-BREF was developed by the WHOQOL Group of the World Health Organization in 1996 [40].

### 2.4. Statistical Analyses

A statistical analysis was performed employing Grand Prism 5 (San Diego, CA, USA) software. Data have been presented as means ± standard deviations (SDs), percentages (%), or 95% confidence intervals (CIs). To carry out the comparisons of psychological variables between the different levels of neuroticism, we calculated the percentiles of the N subscale of the NEO in our sample, as suggested in the literature [41]. We then identified a low level of neuroticism (score = 11; 30th percentile), a medium level (score between 12 and 17; 31st to 69th percentile), and a high level (score = 18; 70th percentile) [41]. Continuous data were compared using the analysis of variance (ANOVA) methods (F) for repeated measures, and categorical data using contingency tables (χ^2^). Bonferroni correction was used to correct for multitesting. Finally, a multivariate logistic regression model of factors associated with higher levels of neuroticism at intake [yielding odds ratios (OR) and their 95% confidence intervals (CIs)] was carried out, with stepwise inclusion of factors in order of their strength (*p*-value) of preliminary bivariate association with neuroticism. Findings with two-tailed *p* ≤ 0.05 were considered statistically significant.

## 3. Results

2630 women, aged 32.4 ± 5.45 years old, were enrolled during their third trimester of pregnancy. Women accessing the Units of Gynecology from July 2020 to November 2022 were consecutively recruited and assessed. Their sociodemographic and clinical characteristics at intake (T0) were collected and are reported in Table 1.

Regarding their education levels, 18 women (0.68%) reported a primary education, 295 (11.23%) secondary education; 1236 (47.03%) post-secondary education; and 1079 (41.06%) higher education. Most of the participants reported being currently employed (n = 1499; 57.17%). Self-reported previous psychiatric disorders in the 6 months preceding pregnancy ranged as follows: 279 women (10.61%) with anxiety disorder > 77 (2.92%) with other disorders > 52 (1.98%) with mood disorders > 35 (1.33%) with eating disorders > 0 (0.00%) with substance abuse > 0 (0.00%) with alcohol abuse. A total of 2187 women (83.16%) reported no previous mental disorders in their personal history.

Table 2 reports the psychological variables as assessed at intake (T0). The general EPDS mean score in the sample was 6.41 ± 4.70, describing a general level of depressive symptoms below the significant cut-off. In the NEO Five-Factor Inventory, neuroticism scored 14.5 ± 7.44, which is considered a low level of neuroticism in the general population. Anxiety and avoidance in close relationships, assessed using the ECR, scored 41.3 ± 18.7 and 29.7 ± 13.8, respectively, confirming low mean levels of both in the sample. Among protective factors for perinatal depression, personal resilience was measured with the CD-RISC assessment and scored 79.0 ± 13.7, describing a medium-to-low level of resilience in the sample. Coping strategies were explored through the Brief-COPE and scored as follows: 6.16 ± 1.47 for the tendency to use adaptive coping strategies such as positive reinterpretation and growth; 5.35 ± 1.61 for self-distraction; 4.99 ± 1.63 for focusing on and venting of emotions; 5.45 ± 1.62 for the use of informational support; 6.75 ± 1.34 for active coping; 3.24 ± 1.43 for denial; 4.86 ± 2.03 for religion; 4.24 ± 1.36 for humor; 2.88 ± 1.22 for behavioral disengagement; 4.98 ± 1.67 for emotional support; 2.07 ± 0.48 for substance use; 6.30 ± 1.34 for acceptance; 6.47 ± 1.46 for planning; and 4.96 ± 1.47 for self-blame. Finally, regarding the quality of life of the participants, as evaluated with the WHOQOL-BREF, the reported total scores for psychological wellbeing (12.2 ± 2.59) and quality of social relationships (12.0 ± 1.91) were both in the normal range.

Of 2630 participants, 2503 women were re-evaluated within 7 days after delivery (T1), with a mean EPDS total score of 5.68 ± 4.05 (slightly lower than the EPDS score at T0). In fact, 127 women dropped-out of the follow-up since they were unavailable for clinical or personal reasons. Subsequently, women considered at risk of depression with EPDS scores ≥ 12, as well as those considered clinically at risk, were reassessed at 1 month and 6 months after delivery. Here we specify that all women reporting EPDS score ≥ 12 at T1 were clinically and properly interviewed by the team of clinicians involved in the study and, when deemed worthy of clinical attention, were selected for subsequent follow-up (T2; N = 488). The number of women reassessed at T3 (N = 456) was lower because a modest number of them dropped-out. Table 3 shows the mean scores registered on the EPDS in the repeated follow-ups over time (T0, T1, T2, and T3).

Table 4 reports the comparison of the EPDS scores of women grouped by their level of neuroticism (low/medium/high) at different times (T0, T1, T2, T3). Specifically, women with high levels of neuroticism reported higher levels of depressive symptoms at baseline, within 7 days of delivery, at 1 month (*all p* < 0.0001), and 6 months after childbirth (*p* = 0.0037).

Comparing the psychological variables of groups with different levels of neuroticism, we found statistically significant differences for all measurements, except the Brief-COPE—Religion scale, with higher scores in women reporting high neuroticism traits (Table 5). Specifically, higher levels of anxiety and avoidance in partner relationships were found in the high-neuroticism group (*p* < 0.0001). In addition, more coping strategies based on positive reinterpretation and growth, focusing on and venting of emotions, use of informational support, denial, behavioral disengagement, emotional support, substance use, and self-blame (*all p* < 0.0001) were found in the high-neuroticism group, with less use of acceptance, active coping, and humor (*all p* < 0.0001). In contrast, there were no significant differences between the medium- and high-neuroticism groups in terms of self-distraction and planning strategies, although there were significant differences in the low-neuroticism group, who made less use of self-distraction (*p* < 0.0001) and more use of planning (*p* < 0.0001). Likewise, lower levels of resilience were found among women with high levels of neuroticism (*p* < 0.0001), among whom lower levels of quality of life in terms of psychological well-being (*p* < 0.0001) and social relationships (*p* < 0.0001) were also found using the WHOQOL scale.

Finally, considering those factors preliminarily associated with the neuroticism levels in the bivariate analyses (Table 4 and Table 5), we performed subsequent logistic multivariate modeling in order to detect those factors associated with higher levels of neuroticism at intake [yielding odds ratios (OR) and their 95% confidence intervals (CIs)]. Characteristics that remained significantly and independently associated were (in descending order of statistical significance): (*a*) higher scores on the EPDS; (*b*) higher scores for ECR Anxiety; (*c*) lower levels of psychological well-being (WHOQOL); (*d*) lower scores on the CD-RISC; (*e*) higher levels of self-blame (Brief-COPE); (*f*) lower levels of active coping (Brief-COPE) (Table 6).

## 4. Discussion

The aim of this study was to investigate the associations between different psychological characteristics and levels of neuroticism among women assessed in their third trimester of pregnancy and followed-up postpartum. The findings confirmed our hypothesis that neuroticism is significantly associated with higher risk of depressive symptoms during both pregnancy and the postpartum period. We further hypothesized that women with higher levels of neuroticism could have presented more anxious and avoidant attachments to their partners, lower quality of life, and more dysfunctional coping strategies with lower levels of resilience. These characteristics were all confirmed by the findings within our sample. The overall mean score for neuroticism in the sample was 14.5 ± 7.44, representing low levels in the general population [15]. We also compared a set of psychological variables within the three groups identified in our sample: low level of neuroticism (score = 11; 30th percentile), medium level (score between 12 and 17; 31st to 69th percentile) and high level (score = 18; 70th percentile). The comparisons revealed that women with high scores for neuroticism reported higher levels of depressive symptoms (EPDS) at baseline (T0), 7 days after delivery (T1), at 1 month and at 6 months postpartum (T2 and T3). In addition, the findings showed statistically significant differences in the psychological measures, highlighting a disadvantage in the relationships, quality of life, coping strategies, and resilience among those women reporting high levels of neuroticism. Our findings were in line with other works that have provided evidence that neuroticism plays an important role in the onset of depressive symptoms and greatly contributes to individual vulnerability to affective disorders [42,43]. In fact, a wide body of international literature has described neuroticism as a predisposing factor for major depression and anxiety disorders [44], for more stressful experience of life events, and for higher reactivity to the effects of adversity [45]. These studies also suggest that the relationship between neuroticism and depression may be even more significant during pregnancy and after childbirth, two periods particularly impacting on woman’s health from a hormonal and emotional point of view [46]. A recent systematic review and meta-analysis indicated that higher levels of neuroticism were associated with an increased risk of postpartum depression [21] and suicidal ideation in the perinatal period [47,48]. Our study aimed to test these associations in a large real-world setting, even if the risk of suicide was clinically detected but not measured in a standardized manner. Iliadis et al. [18] found that pregnant women with high traits of neuroticism reported a four-times higher risk of depressive symptoms after delivery, even after controlling for concurrent factors. In their subsequent study [49], the authors suggested that traits of neuroticism measured at 32 weeks of pregnancy might mediate the association between the familiar vulnerability and the occurrence of depressive symptoms in the postpartum period. Regarding our secondary hypotheses, we found significantly higher levels of anxiety and avoidance in the attachment to partners among women with higher levels of neuroticism. Similarly, many authors found that higher levels of neuroticism were associated with a more insecure attachment and, particularly, that neuroticism was positively related to anxious attachment, worry, and seeking reassurance in relationships [25,50]. Furthermore, we found more dysfunctional coping strategies like denial, behavioral disengagement, substance use, and self-blame in the high-neuroticism group, with less use of acceptance, active coping, and humor. These results are in line with the international literature reporting that neuroticism is associated with several maladaptive behaviors, including low sense of personal control, low self-efficacy, and dysfunctional coping strategies [51,52]. Similarly, lower levels of resilience and poorer quality of life (assessed in terms of psychological well-being and social relationships) were found among women with high levels of neuroticism in our sample. Recently, other authors have also described a negative relationship between resilience and neuroticism [26]. In support of this finding, it has been reported that neuroticism has acted as a predictor of lower emotional resilience during the COVID-19 pandemic in the general population [53]. Our findings regarding the impact on quality of life were also confirmed in a recent large meta-analysis showing that neuroticism is negatively associated with several components of emotional well-being, including quality of life [54]. Similarly, neuroticism seemed to impact on a poor quality of life in various types of clinical samples (e.g., patients with dry eye disease and different types of physical and mental disabilities) [55,56].

The multivariate logistic modeling in our study confirmed that higher EPDS scores, higher ECR Anxiety scores, lower levels of psychological well-being and resilience, as well as dysfunctional coping strategies (e.g., self-blame), all remained significantly and independently associated with higher levels of baseline neuroticism (T0). Thus, in line with other authors, we may argue that neuroticism represents a putative risk factor for mental disorders during pregnancy and postpartum. As already discussed, it represents a stable personality trait predisposing individuals to a greater emotional response to stress as well as greater experience of negative emotions, leading to mental and physical disorders and more frequent attendance at health services [9]. Thus, during pregnancy and after childbirth, women with high levels of neuroticism may report not only higher rates of depressive symptoms, but also worse pregnancy outcomes [57,58]. Finally, in support of our findings, the study conducted by Han et al. confirmed that neuroticism reduces the individual sense of satisfaction and perceived social support [59]. Women with higher levels of neuroticism are inclined to seek both emotional and instrumental support, as well as being less satisfied with their interpersonal relationships, probably suggesting they may perceive this help as unsatisfactory [31].

### Limitations and Strength Points

The limitations of this study may include sampling from the south of Italy only (all the women were from Puglia, Italy) as well as the lack of a longer follow-up (later than six months from delivery) that might have been more informative and more representative of postpartum outcomes. We employed a set of self-report instruments to assess depressive symptoms and other psychological variables. This may be considered a limitation since self-reporting potentially underestimates the variables. Nonetheless, all the instruments used are highly standardized, reliable, and specifically validated. The lack of assessment during the first trimester of pregnancy may be another limitation, even if the international guidance specifically recommends screening for depression during the last trimester. The level of education in the sample was high, and this might have affected the results, even if the sampling was not biased by the inclusion of any educational criteria. Also, cultural aspects were not included in the screening, and this did not allow us to generalize findings internationally. Finally, the mean scores we found using the employed instruments were low, which may suggest that the sample represented a relatively health female population.

The strengths of our study may include the multicenter design, the large sample involved, and the immediate clinical implications of our findings. In fact, after any assessment, women with a high risk of depressive symptoms were properly referred to psychological and mental health services. However, even when referred in the course of a follow-up, they completed the reassessments at T2 and T3. We could not report regarding the effect of these interventions on the reassessment since the number of women referred was low and interventions were not comparable (started with a different timing and individually tailored).

Further studies on a broader population might better explore the role of neuroticism on heterogeneous samples (e.g., including different levels of education, different levels of economic income, etc.). These limitations surely encourage broader recruitment, beyond provincial hospitals, such as from peripheral hospitals and family clinics.

## 5. Conclusions

Identifying risks of depression during pregnancy and postpartum is essential, mainly to prevent adverse health outcomes for the mother and child [60,61,62]. The results of our study indicate that pregnant women may show high vulnerability for affective disorders and should be properly screened and assessed in the course of the pregnancy as well as postpartum. In particular, those women reporting a baseline high level of neuroticism may show disadvantaged psychological characteristics, leading to a higher probability of developing depressive symptoms in the course of their pregnancy. Our protocol also proposed a detailed panel of assessment to be adopted for an early detection of depression and vulnerability factors in the perinatal period. Similarly, this study may provide a set of effective tools for the early referral of women at risk of mood disorders to the available mental health services, as appropriate.

## Figures and Tables

**Table 1:** Socio-demographic and clinical characteristics at intake (T0; N = 2630).

Characteristics		Means ± SD *or* n/% (N = 2630)
Current age (years old)		32.4 ± 5.45
Education, n (%) *	Primary	18 (0.68%)
	Secondary	295 (11.2%)
	Post-secondary	1236 (47.0%)
	Higher education	1079 (41.0%)
Marital status, n (%)	Single	28 (1.06%)
	Widower	0 (0.00%)
	Separated	8 (0.30%)
	Divorced	2 (0.08%)
	Married	1704 (64.7%)
	Engaged	792 (30.1%)
	Coupled	96 (3.65%)
Employment, n (%) **	Yes	1499 (57.1%)
	No	1123 (42.8%)
Previous psychological or mental disorders (6 months), n (%)	None	2187 (83.16%)
	Mood disorders	52 (1.98%)
	Eating disorders	35 (1.33%)
	Substance abuse	0 (0.00%)
	Anxiety disorder	279 (10.61%)
	Alcohol abuse	0 (0.00%)
	Psychosis	0 (0.00%)
	Other	77 (2.92%)

* 2 missing subjects/N = 2630; ** 8 missing subjects/N = 2630.

**Table 2:** Psychological characteristics at intake (T0; N = 2630).

Characteristics	Means ± SDs
EPDS	6.41 ± 4.70
NEO-FFI	14.5 ± 7.44
ECR *Anxiety*	41.3 ± 18.7
ECR *Avoidance*	29.7 ± 13.8
CD-RISC	79.0 ± 13.7
Brief COPE—*Positive reinterpretation and growth*	6.16 ± 1.47
*Brief COPE*—*Self-distraction*	5.35 ± 1.61
*Brief COPE*—*Focusing on and venting of emotions*	4.99 ± 1.63
*Brief COPE*—*Use of informational support*	5.45 ± 1.62
*Brief-COPE*—*Active coping*	6.75 ± 1.34
*Brief-COPE*—*Denial*	3.24 ± 1.43
*Brief-COPE*—*Religion*	4.86 ± 2.03
*Brief COPE*—*Humor*	4.24 ± 1.36
*Brief COPE*—*Behavioral disengagement*	2.88 ± 1.22
*Brief-COPE*—*Emotional support*	4.98 ± 1.67
*Brief-COPE*—*Substance use*	2.07 ± 0.48
*Brief-COPE*—*Acceptance*	6.30 ± 1.34
*Brief-COPE*—*Planning*	6.47 ± 1.46
*Brief-COPE*—*Self-blame*	4.96 ± 1.47
WHOQOL—*Psychological wellbeing *	12.2 ± 2.59
*WHOQOL*—*Social relations*	12.0 ± 1.91

Note: EPDS = Edinburgh Postnatal Depression Scale; NEO-FFI = the N scale of the 60 items NEO Five-Factor Inventory; ECR = the Experience in Close Relationship scale; CD-RISC = the Connor—Davidson Resilience Scale; the Brief-COPE= coping strategies;the WHOQOL = the World Health Organization Quality of Life.

**Table 3:** EPDS scores at intake (T0; N = 2630) and follow-up times. (T1, N = 2503; T2, N = 488; T3, N = 456).

Variables	Means ± SDs
EPDS T0 (N = 2630)	6.41 ± 4.70
EPDS T1 (N = 2503)	5.68 ± 4.05
EPDS T2 (N = 488)	6.41 ± 4.23
EPDS T3 (N = 456)	5.60 ± 4.11

Note: EPDS = Edinburgh Postnatal Depression Scale; T1 = 7 days after childbirth; T2 = 1 month; T3 = 6 months.

**Table 4:** Associations between EPDS scores and levels of neuroticism (low-, middle-, high-levels/percentiles) at intake (T0; N = 2630) and follow-up times (T1, N = 2503; T2, N = 488; T3, N = 456).

Variables	Low Level of Neuroticism	Medium Level of Neuroticism	High Level of Neuroticism	*F*	*p*-Value
EPDS T0	3.82 ± 3.21	5.8 ± 3.78	9.88 ± 4.90	713.7	<0.0001
EPDS T1	4.05 ± 3.11	5.33 ± 3.61	7.81 ± 4.19	412.3	<0.0001
EPDS T2	5.31 ± 3.72	6.32 ± 3.88	7.59 ± 4.67	24.15	<0.0001
EPDS T3	4.88 ± 3.66	5.74 ± 3.76	6.12 ± 4.29	11.21	0.0037

Note: EPDS = Edinburgh Postnatal Depression Scale; neuroticism = measured with the N scale of the 60-items NEO Five-Factor Inventory (60-items NEO-FFI); T1 = 7 days after childbirth; T2 = 1 month postpartum; T3 = 6 months postpartum.

**Table 5:** Associations between psychological variables and levels of neuroticism at intake (T0; N = 2630).

Variables	Low Level of Neuroticism (n = 886)	Medium Level of Neuroticism (n = 918)	High Level of Neuroticism (n = 825)	*F*	*p*-Value
ECR *Anxiety*	32.4 ± 13.3	38.1 ± 15.4	54.4 ± 20.0	410	<0.0001
*Avoidance*	24.8 ± 9.04	28.6 ± 12.0	36.3 ± 16.9	174	<0.0001
CD-RISC	84.7 ± 10.1	79.6 ± 13.1	72.1 ± 14.8	208	<0.0001
Brief COPE— *Positive reinterpretation and growth*	6.47 ± 1.38	6.15 ± 1.46	6.84 ± 1.50	40.5	<0.0001
*Self-distraction*	5.15 ± 1.65	5.39 ± 1.56	5.53 ± 1.57	12.1	<0.0001
*Focusing on and venting of emotions*	4.84 ± 1.82	4.82 ± 1.48	5.31 ± 1.52	24.8	<0.0001
*Use of informational support*	5.29 ± 1.59	5.36 ± 1.64	5.69 ± 1.59	14.9	<0.0001
*Active coping*	7.08 ± 1.21	6.66 ± 1.43	6.48 ± 1.28	46.8	<0.0001
*Denial*	2.78 ± 1.13	3.23 ± 1.34	3.74 ± 1.16	105.1	<0.0001
*Religion*	4.83 ± 2.03	4.86 ± 2.02	4.88 ± 2.04	0.09	0.9842
*Humor*	4.50 ± 1.30	4.22 ± 1.33	3.97 ± 1.39	33.18	<0.0001
*Behavioral disengagement*	2.41 ± 0.81	2.83 ± 1.15	3.41 ± 1.41	163.7	<0.0001
*Emotional support*	4.71 ± 1.55	4.85 ± 1.68	5.43 ± 1.69	46.4	<0.0001
*Substance use*	2.04 ± 0.31	2.03 ± 0.34	2.14 ± 0.7	12.61	<0.0001
*Acceptance*	6.54 ± 1.23	6.24 ± 1.38	6.11 ± 1.35	24.17	<0.0001
*Planning*	6.87 ± 1.29	6.32 ± 1.53	6.18 ± 1.43	57.31	<0.0001
*Self-blame*	4.69 ± 1.32	4.78 ± 1.48	5.43 ± 1.47	67.43	<0.0001
WHOQOL-BREF—*Psychological wellbeing*	13.3 ± 2.19	12.5 ± 2.30	10.7 ± 2.53	286.4	<0.0001
*Social relations*	12.6 ± 1.68	12.2 ± 1.79	11.2 ± 1.97	144.4	<0.0001

Note: ECR = the Experience in Close Relationship scale; CD-RISC = the Connor—Davidson Resilience Scale; the Brief-COPE; the WHOQOL-BREF = World Health Organization Quality of Life; Bonferroni correction for multiple testing was employed.

**Table 6:** Multivariate logistic regression model of factors associated with higher levels of Neuroticism at intake (T0; N = 2630).

Factors	Slope (β-Coeff)	OR [95% CI]	*t*-Score	*p*-Value
Higher scores on the EPDS	3.65	[0.43–0.98]	7.49	<0.001
Higher scores for ECR Anxiety	1.78	[1.02–1.78]	13.2	<0.001
Lower levels of psychological well-being (WHOQOL-BREF)	−2.34	[0.35–0.78]	8.75	0.0045
Lower scores on the CD-RISC	−2.13	[1.06–2.01]	11.3	0.0056
Higher levels of self-blame (Brief-COPE)	3.54	[0.56–0.77]	6.35	0.0467
Lower levels of active coping (Brief-COPE)	−3.22	[0.23–0.84]	8.45	0.0471

Note: EPDS = Edinburgh Postnatal Depression Scale; ECR = the Experience in Close Relationship scale; CD-RISC = the Connor—Davidson Resilience Scale; the Brief-COPE; the WHOQOL-BREF = World Health Organization Quality of Life.

## Data Availability

The raw data supporting this article will be made available by the authors, upon request. The data are not publicly available due to sensitive information contained.

## References

[1] San Martin Porter M.A., Kisely S., Salom C., Betts K.S., Alati R. (2022). Association between screening for antenatal depressive symptoms and delivery outcomes: The Born in Queensland Study. Aust. N. Z. J. Obstet. Gynaecol..

[2] Reilly N., Kingston D., Loxton D., Talcevska K., Austin M.-P. (2020). A narrative review of studies addressing the clinical effectiveness of perinatal depression screening programs. Women Birth.

[3] Siu A.L., US Preventive Services Task Force (2016). Screening for depression in adults: US Preventive Services Task Force recommendation statement. JAMA.

[4] Austin M.-P., Highet N., The Expert Working Group (2018). Mental Health Care in the Perinatal Period: Australian Clinical Practice Guideline. Melbourne: Centre of Perinatal Excellence. http://cope.org.au/wp-content/uploads/2018/05/COPE-Perinatal-MH-Guideline_Final-2018.pdf.

[5] Podolska M.Z., Majkowicz M., Bidzan M., Pankiewicz P., Sipak-Szmigiel O., Podolski J. (2010). Increased neuroticism aggravates the risk of depressiye symptoms pregnant women. Stud. Psychol..

[6] Podolska M.Z., Bidzan M., Majkowicz M., Podolski J., Sipak-Szmigiel O., Ronin-Walknowska E. (2010). Personality traits assessed by the NEO Five-Factor Inventory (NEO-FFI) as part of the perinatal depression screening program. Med. Sci. Monit..

[7] Meuti V., Marini I., Grillo A., Lauriola M., Leone C., Giacchetti N., Aceti F. (2014). MMPI-2: Cluster analysis of personality profiles in perinatal depression—Preliminary evidence. Sci. World J..

[8] Sun J.W., Li J.H., Zhang X., Wang Y., Cao D.F., Wang J., Bai H.Y., Lin P.Z., Zhang H.H., Sun Y.Y. (2020). Perinatal depression: Data-driven subtypes derived from life history and mindfulness and personality. J. Affect. Disord..

[9] Bellomo A., Severo M., Petito A., Nappi L., Iuso S., Altamura M., Marconcini A., Giannaccari E., Maruotti G., Palma G.L. (2022). Perinatal depression screening and prevention: Descriptive findings from a multicentric program in the South of Italy. Front. Psychiatry.

[10] Altamura M., Leccisotti I., De Masi L., Gallone F., Ficarella L., Severo M., Biancofiore S., Denitto F., Ventriglio A., Petito A. (2023). Coping as a Mediator between Attachment and Depressive Symptomatology Either in Pregnancy or in the Early Postpartum Period: A Structural Equation Modelling Approach. Brain Sci..

[11] Ventriglio A., Severo M., Petito A., Nappi L., Iuso S., Altamura M., Sannicandro V., Milano E., Arcidiacono G., Di Salvatore M. (2023). The impact of body mass index on the pregnancy outcomes and risk of perinatal depression: Findings from a multicenter Italian study. Eur. Psychiatry.

[12] Eysenck H.J. (1967). The Biological Basis of Personality.

[13] Lahey B.B. (2009). Public health significance of neuroticism. Am. Psychol..

[14] Hettema J.M., Neale M.C., Myers J.M., Prescott C.A., Kendler K.S. (2006). A population-based twin study of the relationship between neuroticism and internalizing disorders. Am. J. Psychiatry.

[15] McCrae R., Costa P. (2004). A contemplated revision of the NEO five-factor inventory. Pers. Individ. Differ..

[16] Roelofs J., Huibers M., Peeters F., Arntz A., van Os J. (2008). Rumination and worrying as possible mediators in the relation between neuroticism and symptoms of depression and anxiety in clinically depressed individuals. Behav. Res. Ther..

[17] Barlow D.H., Sauer-Zavala S., Carl J.R., Bullis J.R., Ellard K.K. (2014). The nature, assessment, and treatment of neuroticism: Back to the future?. Clin. Psychol. Sci..

[18] Dennis C.L.E., Janssen P.A., Singer J. (2004). Identifying women at-risk for postpartum depression in the immediate postpartum period. Acta Psychiatr. Scand..

[19] Puyané M., Subirà S., Torres A., Roca A., Garcia-Esteve L., Gelabert E. (2022). Personality traits as a risk factor for postpartum depression: A systematic review and meta-analysis. J. Affect. Disord..

[20] Iliadis S.I., Koulouris P., Gingnell M., Sylvén S.M., Sundström-Poromaa I., Ekselius L., Papadopoulos F.C., Skalkidou A. (2015). Personality and risk for postpartum depressive symptoms. Arch. Women’s Ment. Health.

[21] Bright A.M., Doody O., Tuohy T. (2022). Women with perinatal suicidal ideation-A scoping review of the biopsychosocial risk factors to inform health service provision and research. PLoS ONE.

[22] Swickert R., Hitner J.B., Foster A. (2010). Big Five traits interact to predict perceived social support. Pers. Individ. Dif..

[23] Johnston R.G., Brown A.E. (2013). Maternal trait personality and childbirth: The role of extraversion and neuroticism. Midwifery.

[24] Chen J., Sun M., Huang C., Xiao J., Tang S., Chen Q. (2022). Pathways from Neuroticism, Social Support, and Sleep Quality to Antenatal Depression during the Third Trimester of Pregnancy. Int. J. Environ. Res. Public Health.

[25] Noftle E.E., Shaver P.R. (2006). Attachment dimensions and the big five personality traits: Associations and comparative ability to predict relationship quality. J. Res. Pers..

[26] Nieto M., Visier M.E., Silvestre I.N., Navarro B., Serrano J.P., Martínez-Vizcaíno V. (2023). Relation between resilience and personality traits: The role of hopelessness and age. Scand. J. Psychol..

[27] Aarstad A.K., Beisland E., Osthus A.A., Aarstad H.J. (2011). Distress, quality of life, neuroticism and psychological coping are related in head and neck cancer patients during follow-up. Acta Oncol..

[28] Karimzade A., Ali Besharat M. (2011). An investigation of the relationship between personality dimensions and stress coping styles. Procedia Soc. Behav. Sci..

[29] World Medical Association (2013). World Medical Association declaration of Helsinki ethical principles for medical research involving human subjects. J. Am. Med. Assoc..

[30] Yang K., Wu J., Chen X. (2022). Risk factors of perinatal depression in women: A systematic review and meta-analysis. BMC Psychiatry.

[31] Kofman Y.B., Eng Z.E., Busse D., Godkin S., Campos B., Sandman C.A., Wing D., Yim I.S. (2019). Cortisol reactivity and depressive symptoms in pregnancy: The moderating role of perceived social support and neuroticism. Biol. Psychol..

[32] Cox J.L., Holden J.M., Sagovsky R. (1987). Detection of postnatal depression. Development of the 10-item Edinburgh Postnatal Depression Scale. Br. J. Psychiatry.

[33] Benvenuti P., Ferrara M., Niccolai C., Valoriani V., Cox J.L. (1999). The Edinburgh Postnatal Depression Scale: Validation for an Italian sample. J. Affect. Disord..

[34] Brennan K.A., Clark C.L., Shaver P.R., Simpson J.A., Rholes W.S. (1998). Self-report measurement of adult attachment: An integrative overview. Attachment Theory and Close Relationships.

[35] Picardi A., Vermigli P., Toni A., D’Amico R., Bitetti D., Pasquini P. (2002). Il questionario “experiences in close relationships” (ECR) per la valutazione dell’attaccamento negli adulti: Ampliamento delle evidenze di validità per la versione italiana. Ital. J. Psychopathol..

[36] Connor K.M., Davidson J.R. (2003). Development of a new resilience scale: The Connor-Davidson resilience scale (CD-RISC). Depress. Anxiety.

[37] Carver C.S., Scheier M.F., Krohne H.W. (1993). Vigilant and avoidant coping in two patient samples. Attention and Avoidance: Strategies in Coping with Aversiveness.

[38] Conti L. (2000). Repertorio Delle Scale di Valutazione in Psichiatria.

[39] de Girolamo G., Rucci P., Scocco P., Becchi A., Coppa F., D’Addario A., Daru E., De Leo D., Galassi L., Mangelli L. (2000). La valutazione della qualità della vita: Validazione del WHOQOL-Breve [Quality of life assessment: Validation of the Italian version of the WHOQOL-Brief]. Epidemiol. Psichiatr. Soc..

[40] World Health Organization (1996). Division of mental health. WHOQOL-BREF: Introduction, Administration, Scoring and Generic Version of the Assessment: Field Trial Version.

[41] Wilson R.S., Krueger K.R., Gu L., Bienias J.L., Mendes de Leon C.F., Evans D.A. (2005). Neuroticism, extraversion, and mortality in a defined population of older persons. Psychosom. Med..

[42] Vittengl J.R. (2017). Who pays the price for high neuroticism? Moderators of longitudinal risks for depression and anxiety. Psychol. Med..

[43] Jirakran K., Vasupanrajit A., Tunvirachaisakul C., Maes M. (2023). The effects of adverse childhood experiences on depression and suicidal behaviors are partially mediated by neuroticism: A subclinical manifestation of major depression. Front. Psych..

[44] Axfors C., Eckerdal P., Volgsten H., Wikström A.H., Ekselius L., Ramklint M., Sundström Poromaa I., Skalkidou A. (2019). Investigating the association between neuroticism and adverse obstetric and neonatal outcomes. Sci. Rep..

[45] Kendler K.S., Kuhn J., Prescott C.A. (2004). The interrelationship of neuroticism, sex, and stressful life events in the prediction of episodes of major depression. Am. J. Psychiatry.

[46] Roman M., Bostan C.M., Diaconu-Gherasim L.R., Constantin T. (2019). Personality Traits and Postnatal Depression: The Mediated Role of Postnatal Anxiety and Moderated Role of Type of Birth. Front. Psychol..

[47] Duan C., Hare M.M., Staring M., Deligiannidis K.M. (2019). Examining the relationship between perinatal depression and neurodevelopment in infants and children through structural and functional neuroimaging research. Int. Rev. Psychiatry.

[48] Gelabert E., Gutierrez-Zotes A., Navines R., Labad J., Puyane M., Donadon M., Guillamat R., Mayoral F., Jover M., Canellas F. (2020). The role of personality dimensions, depressive symptoms and other psychosocial variables in predicting postpartum suicidal ideation: A cohort study. Arch. Womens Ment. Health.

[49] Iliadis S.I., Comasco E., Hellgren C., Kollia N., Sundström Poromaa I., Skalkidou A. (2017). Associations between a polymorphism in the hydroxysteroid (11-beta) dehydrogenase 1 gene, neuroticism and postpartum depression. J. Affect. Disord..

[50] Anagnostopoulos F., Botse T. (2016). Exploring the Role of Neuroticism and Insecure Attachment in Health Anxiety, Safety-Seeking Behavior Engagement, and Medical Services Utilization: A Study Based on an Extended Interpersonal Model of Health Anxiety. SAGE Open.

[51] Szymura B., Saklofske D.H., Zeidner M. (2010). Individual differences in resource allocation policy. Handbook of Individual Differences in Cognition.

[52] de la Fuente J., Paoloni P., Kauffman D., Yilmaz Soylu M., Sander P., Zapata L. (2020). Big Five, Self-Regulation, and Coping Strategies as Predictors of Achievement Emotions in Undergraduate Students. Int. J. Environ. Res. Public Health.

[53] Gonneaud J., Paly L., Delarue M., Mézenge F., Fauvel S., Lefranc V., Cognet A., de Flores R., Touron E., Marchant N.L. (2021). Neuroticism is the best predictor of lower emotional resilience during the COVID-19-related confinement periods. Alzheimer’s Dement..

[54] DeNeve K.M., Cooper H. (1998). The happy personality: A meta-analysis of 137 personality traits and subjective well-being. Psychol. Bull..

[55] Tananuvat N., Tansanguan S., Wongpakaran N., Wongpakaran T. (2022). Role of neuroticism and perceived stress on quality of life among patients with dry eye disease. Sci. Rep..

[56] Cai L., He J., Wu Y., Jia X. (2023). The relationship between big five personality and quality of life of people with disabilities: The mediating effect of social support. Front. Psychol..

[57] Chatzi L., Koutra K., Vassilaki M., Vardiampasis A., Georgiou V., Koutis A., Lionis C., Bitsios P., Kogevinas M. (2013). Maternal personality traits and risk of preterm birth and fetal growth restriction. Eur. Psychiatry.

[58] Finch J.F., Graziano W.G. (2001). Predicting depression from temperament, personality, and patterns of social relations. J. Pers..

[59] Han J., Leng X., Gu X., Li Q., Wang Y., Chen H. (2021). The role of neuroticism and subjective social status in the relationship between perceived social support and life satisfaction. Personal. Individ. Differ..

[60] Atif N., Lovell K., Rahman A. (2015). Maternal mental health: The missing “m” in the global maternal and child health agenda. Semin. Perinatol..

[61] Severo M., Ventriglio A., Bellomo A., Iuso S., Petito A. (2023). Maternal perinatal depression and child neurocognitive development: A relationship still to be clarified. Front. Psychiatry.

[62] Fitzgerald L., McNab S., Njau P., Chandra P., Koyiet P., Levine R., Hardtman P., Stalls S. (2024). Beyond survival: Prioritizing the unmet mental health needs of pregnant and postpartum women and their caregivers. PLOS Glob. Public Health.

